# Nonsmokers’ responses to new warning labels on smokeless tobacco and electronic cigarettes: an experimental study

**DOI:** 10.1186/1471-2458-14-997

**Published:** 2014-09-25

**Authors:** Lucy Popova, Pamela M Ling

**Affiliations:** Center for Tobacco Control Research and Education, University of California San Francisco, 530 Parnassus Avenue, Suite 366, San Francisco, CA 94143 USA; Division of General Internal Medicine, Department of Medicine, University of California San Francisco, San Francisco, USA

**Keywords:** Tobacco, Smokeless tobacco, Electronic cigarette, Warning labels, Snus

## Abstract

**Background:**

Graphic warning labels are a tobacco control best practice that is mandated in the US for cigarettes under the 2009 Family Smoking Prevention and Tobacco Control Act. However, smokeless tobacco products are not required to carry graphic warning labels, and as of September 2014, electronic cigarettes in the US carry no warning labels and are aggressively marketed, including with “reduced harm” or “FDA Approved” messages.

**Methods:**

In this online experiment, 483 US adult non-users of tobacco were randomized to view print advertisements for moist snuff, snus, and e-cigarettes with either warning labels (current warning label, graphic warning label) or “endorsements” (a “lower risk” label proposed by a tobacco company, an “FDA Approved” label) or control (tobacco advertisement with no label, advertisement for a non-tobacco consumer products). Main outcome measures included changes in perceived harm, positive attitudes towards, openness to using, and interest in a free sample of moist snuff, snus, and e-cigarettes.

**Results:**

The graphic warning label increased perceived harm of moist snuff and e-cigarettes. “Lower risk” and “FDA Approved” labels decreased perceived harm of moist snuff and snus respectively. Current warning label and graphic warning label significantly lowered positive attitudes towards e-cigarettes. In this sample of non-users of tobacco, 15% were interested in a free sample of alternative tobacco products (predominantly e-cigarettes). Proportion of participants interested in a free sample did not differ significantly across the conditions, but those interested in a free sample had significantly lower perceptions of harm of corresponding tobacco products.

**Conclusions:**

Regulatory agencies should not allow “lower risk” warning labels, which have similar effects to the “FDA Approved” label, which is prohibited, and should consider implementing graphic warning labels for smokeless tobacco products and e-cigarettes.

## Background

Tobacco use remains the leading cause of preventable death in the United States [1]. Risks of tobacco use have been identified [2], and understanding of these risks is one of the main factors that explains initiation and cessation of tobacco use [3–7].

One of the central approaches to increasing perceived risk of tobacco products has been the use of warning labels [8]. Most research to date has focused on cigarette warning labels, specifically graphic warning labels, which have been mandated by law in 63 countries globally [9]. Graphic warning labels promote cessation behavior [10, 11] and are effective in communicating the harm of cigarettes [8]. In the US, in 2009 the Family Smoking Prevention and Tobacco Control Act mandated the Food and Drug Administration (FDA) to implement graphic warning labels on cigarettes, bringing the US closer in alignment to the World Health Organization’s Framework Convention on Tobacco Control (FCTC) [12] and the European Commission’s Tobacco Product Directive [13], both of which recommend large pictorial health warnings. The proposed US graphic warning labels would cover top 50% of both front and back of cigarette packages, which is consistent with WHO FCTC recommendation of 50% or more of the principal display areas [12], but is less than the recently revised European Commission’s Tobacco Product Directive (65% of the front and back of the cigarette pack) [13].

In addition, new warning labels may be developed for tobacco products other than cigarettes. In recent years, tobacco and e-cigarette companies have been aggressively developing and promoting new alternative tobacco products, such as electronic cigarettes (e-cigarettes), snus, and dissolvable tobacco products [14–17]. E-cigarettes are electronic devices generally consisting of a battery connected to a heater, a mouthpiece, and a chamber containing a solution of propylene glycol and other chemicals, frequently including nicotine. When the device is used, the solution is vaporized by the heater, producing an aerosol that is inhaled. Snus is ground tobacco placed in a porous pouch to be placed between lip and gum to allow nicotine absorption; snus sold in the US is modeled after a traditional Swedish product which typically is manufactured so that it contains fewer tobacco-specific nitrosamines (carcinogens) than other forms of chewing tobacco [18].

Currently, some of these products (smokeless tobacco) carry warning labels, and others (e-cigarettes) do not. While graphic warning labels have been mandated by law for cigarettes, they have not been established for alternative tobacco products, and research on the effects of graphic warning labels on smokeless tobacco is nascent [19]. There is no research on the effects of warning labels on e-cigarettes, and the current marketing frequently refers to reduced risk. A systematic content analysis of electronic cigarette websites found 95% of websites made health related claims, such as the statement, “Can’t argue with 4,000 less chemicals!” [20]. While it has been suggested that e-cigarettes should be promoted as “reduced harm” alternatives to combustible cigarettes for smokers [21], there are no long term data on the health effects of e-cigarette use, and widespread promotion may also result in uptake among non-users of tobacco, which would be inconsistent with harm reduction on a population level [22]. Warning labels on e-cigarettes might discourage people who do not use tobacco from starting. We investigated the effects of placing warning labels on e-cigarette advertising on perceived harm of e-cigarettes among non-users, hypothesizing that any warning label will increase perceptions of harm of e-cigarettes.

Since 1998, marketing expenditures for smokeless tobacco have increased by 277% [23]. Since 2009, cigarette companies have purchased smokeless tobacco companies, and now sell both traditional and novel smokeless tobacco products, which are frequently promoted for temporary use in smoke-free environments [14]. In the United States, smokeless tobacco products (moist snuff, snus, dissolvables) currently display one of four text warning labels mandated for smokeless tobacco products:

- “WARNING: This product can cause mouth cancer.”
- “WARNING: This product can cause gum disease and tooth loss.”
- “WARNING: This product is not a safe alternative to cigarettes.”
- “WARNING: Smokeless tobacco is addictive.”

In 2011, R. J. Reynolds Tobacco Company submitted a Citizen Petition to the US Food and Drug Administration requesting to change one of the smokeless tobacco warning labels from “WARNING: This product is not a safe alternative to cigarettes” to “WARNING: No tobacco product is safe, but this product presents substantially lower risks to health than cigarettes” [24], linking the warning statement to an implicit endorsement.

In addition, there have been longstanding concerns that tobacco companies might try to use FDA regulation (and their compliance with it) as a marketing strategy and promote new tobacco products as meeting the FDA requirements or being “FDA Approved”. Although the Family Smoking Prevention and Tobacco Control Act explicitly prohibits use of “FDA Approved” language for marketing of tobacco products, this restriction currently does not apply to e-cigarettes, which are sometimes advertised as “made in an FDA Approved facility” [25–28]. The “endorsement” label proposed by R. J. Reynolds may also be interpreted like an “FDA Approved” label.

To our knowledge, there are currently no studies on the effect of communicating FDA oversight of tobacco on consumer perceptions of harm of new tobacco products. The R. J. Reynolds’ proposed “endorsement” label has not been studied empirically, and it might act equivalently to an “FDA Approved” endorsement. This study compared the label proposed by R. J. Reynolds to a label stating the product is “FDA Approved”. We hypothesized that exposure to either of the reduced harm messages would decrease perceived harm of alternative tobacco products, and that the R. J. Reynolds label would have an effect similar to the “FDA Approved” label.

## Methods

### Participants

Participants were 506 non-users of tobacco recruited by Toluna (http://www.toluna-group.com), a survey and market research company, to complete a single online experiment. Participants were recruited through a variety of online and offline recruitment strategies and responded to surveys in exchange for cash rewards provided by Toluna. The sample was screened to include only adults aged 18+ who were not established tobacco users, (e.g., they had not smoked 100 cigarettes in their entire life, and had not used smokeless tobacco such as chewing tobacco, snuff, dip, or snus at least 20 times in their entire life). Participants who reported current (past 30 day) use of any alternative tobacco products (n = 23) were excluded from further analyses in order to focus on non-users of tobacco. Thus, the final sample included 483 non-users of tobacco. Among them, 4% had ever tried one of the new and alternative tobacco products, but not in the past month.

To ensure eligibility of participants, participants had to enter their zip code in the beginning and end of the survey; those whose zip codes did not match had their session terminated. Additional procedures for data quality control are described at http://www.toluna-group.com/about-toluna/about/data-quality-approach. All participants completed electronic informed consent forms and all protocols were approved by the Committee on Human Research (the IRB) at the University of California San Francisco.

### Procedure

**Figure 1:**
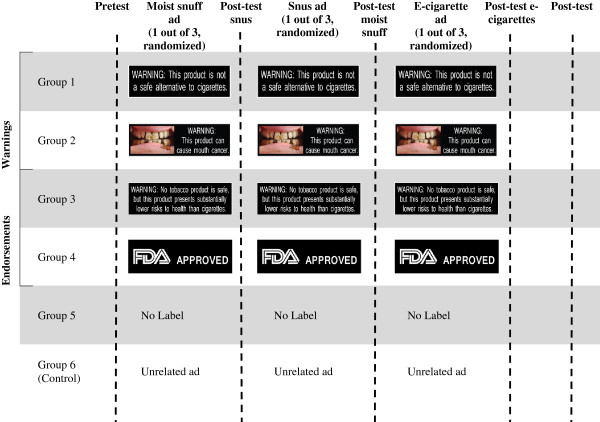
**Study design and experimental procedure.**

The experimental procedure is illustrated in Figure 1. Participants began by filling out a pretest questionnaire comprising demographic questions, measures of past tobacco use and outcome variables (perceived harm, positive attitudes, and openness to using moist snuff, snus, and e-cigarettes). They were then randomized to one of six groups: five groups saw advertisements for alternative tobacco products with either a warning: 1) current warning label, “Warning: This product is not a safe alternative to cigarettes” (this warning is currently mandated for smokeless tobacco), 2) graphic warning label (picture of a mouth sore and words “Warning: This product can cause mouth cancer;” an endorsement: 3) R. J. Reynolds’s proposed “lower risk” label, “Warning: No tobacco product is safe, but this product presents substantially lower risks to health than cigarettes,” 4) “FDA Approved” label; 5) an advertisement for a tobacco product with no warning label, or 6) a control group that saw advertisements for a non-tobacco consumer products (such as a cell phone or gum) (Figure 1). While the “FDA Approved” label is prohibited for tobacco products currently regulated by the FDA, this condition was used to facilitate comparison to the prohibited message. In each experimental condition, participants saw advertisements for three products (presented in random order to mitigate order effects): moist snuff, snus, and e-cigarettes. For each product, the advertisement shown was randomly drawn from three advertisements for each type of alternative tobacco products. Warning labels were added to the bottom of each advertisement to cover 20% of the total area of the advertisement as required by law for smokeless tobacco warnings [29]. Following exposure to each advertisement, participants completed outcome measures (perceived harm, attitudes, and intentions to use this product) in a posttest. Median time to complete the study was 12 minutes.

### Measures

#### Demographics and tobacco use

Demographic variables included sex, age, race, annual household income, educational level, and geographical region. Ever and current (past 30 days) use of alternative tobacco products was measured for each of five alternative tobacco products: loose leaf, moist snuff, snus, dissolvables, and electronic cigarettes.

#### Outcome variables

*Perceived harm* of tobacco products was measured with two questions: ‘In your opinion, how harmful is … (moist snuff, snus, e-cigarettes) to general health?’ and ‘In your opinion, to what extent does … cause cancer?’ reported on 9-point Likert scales ranging from ‘Not at all’ to ‘Extremely.’ The answers to the two questions were averaged to create a perceived harm scale (Cronbach’s alphas 0.95-0.98) for each of the three products. The *positive attitudes* scales comprised four items measured on a 9-point semantic differential scales: “Using (moist snuff, snus, e-cigarettes) is: good-bad, intelligent-unintelligent, appropriate-not appropriate, and pleasant-unpleasant” with higher scores indicating more positive attitudes (Cronbach’s alphas 0.96-0.98). The answers to four questions were averaged. *Openness to trying* each of the alternative tobacco products was measured with one item, “How open are you to trying … (moist snuff, snus, e-cigarettes) in the future?” with answers on 9-point Likert scales ranging from ‘Not at all open’ to ‘Extremely open’.

#### Behavioral task - selection of free sample of alternative tobacco products

After the exposure to each advertisement, participants were offered a free sample of alternative tobacco products and asked to select the brand and flavor. After seeing a moist snuff advertisement, participants could choose among Copenhagen, Grizzly, or Skoal moist snuff. After seeing a snus advertisement, participants could choose among Camel snus, Marlboro snus, and General snus, and after seeing an e-cigarette advertisement, they could choose among blu, V2, or NJOY e-cigarette. In every case, they could also select “Not interested in a free sample” option. In the end of the study, the participants were informed that no samples would actually be mailed to them and that this study did not endorse or promote tobacco use in any way. This behavioral selection task has been used in prior studies [30, 31].

All products in the same experimental condition bore the same warning label. In the end, participants were asked to select which warning label they saw on the advertisements, with choices being “No warning labels”, the text for the four labels (warnings and endorsements) used in the study, and two additional smokeless tobacco warning labels.

### Statistical analyses

All analyses were conducted in IBM SPSS version 21. To examine the effects of individual warning labels on changes in perceived harm we used general linear models (GLM) with time as within-subject factor and warning label condition as between-subject factor. Interactions between time and condition were examined to determine whether any significant omnibus differences over time by condition were present. In addition, we explored pre-post differences within each condition in order to determine which conditions exhibited the strongest differences between the pretest and posttest measurements. Multiple paired comparisons of cell means featured *p*-value adjustment via Tukey’s honestly significant difference (HSD) procedure to maintain a nominal alpha of .05.

## Results

### Description of the sample

The national sample of 483 non-users of tobacco was 44.1% male, mean age was 47 years. There were no significant differences in participant characteristics between experimental conditions (Table 1). Levels of outcome variables (perceived harm, attitudes, and openness to using moist snuff, snus, and e-cigarettes) measured at pretest are presented in Table 2.

**Table 1:** **Participant demographic characteristics for the total sample and for each (randomly assigned) experimental group**

Characteristic	N	%	No warning label (n = 75),%	Current warning label (n = 74),%	Lower risks warning label (n = 75),%	FDA approved warning label (n = 79),%	Graphic warning label (n = 76),%	Control (n = 76),%
Gender								
Male	206	44.1	45.0	38.5	39.2	48.8	50.0	42.5
Female	249	55.9	55.0	61.5	60.8	51.2	50.0	57.5
Age, years								
18-29	70	17.2	13.8	16.7	15.2	22.6	15.9	18.8
30-44	127	27.3	31.3	32.1	27.8	20.2	29.3	23.8
45-59	134	28.8	28.7	19.2	32.9	27.4	26.8	37.5
60+	124	26.7	26.3	32.1	24.1	29.8	28.0	20.0
Race								
White	232	53.0	55.0	46.2	57.0	57.1	53.7	48.8
Black or African American	94	20.1	13.8	25.6	24.1	14.3	20.7	22.5
American Indian or Alaska Native	12	2.7	3.8	3.8	2.5	2.4	1.2	2.5
Asian	91	18.6	23.8	16.7	12.7	19.0	19.5	20.0
Native Hawaiian or Pacific Islander	3	0.8	0.0	2.6	0.0	1.2	1.2	0.0
Multiple Races	21	4.3	2.5	5.1	3.8	6.0	3.7	5.0
Unknown	2	0.4	1.3	0.0	0.0	0.0	0.0	1.3
Education								
High school or less	81	19.7	15.0	20.5	21.5	16.7	20.7	23.8
Some college	91	21.9	22.5	20.5	21.5	25.0	24.4	17.5
Bachelor’s degree or higher	283	58.4	62.5	59.0	57.0	58.3	54.9	58.8
Annual Household Income (thousand USD)								
<25	109	24.6	28.7	33.3	19.0	25.0	19.5	22.5
25-59.9	181	40.0	40.0	32.1	41.8	39.3	47.6	38.8
>60	165	35.4	31.3	34.6	39.2	35.7	32.9	38.8
Region								
Northeast	76	16.4	18.8	12.8	19.0	14.3	13.4	20.0
Midwest	90	20.5	20.0	25.6	26.6	20.2	18.3	12.5
South	172	37.5	37.5	33.3	29.1	46.4	41.5	36.3
West	117	25.7	23.8	28.2	25.3	19.0	26.8	31.3

Note: No significant differences were found between conditions on participant characteristics.

**Table 2:** **Mean (SD) levels of perceived harm, positive attitudes, and openness to trying alternative tobacco products at pretest**

	Perceived harm	Positive attitudes	Openness to trying
**Moist snuff**	7.13 (2.59)	1.72 (1.50)	1.08 (0.57)
**Snus**	7.35 (2.13)	1.88 (1.62)	1.08 (0.50)
**E-cigarettes**	4.91 (2.82)	3.47 (2.59)	1.34 (1.20)

Note: All measures were on 1–9 scales with higher scores indicating greater perceived harm, more positive attitudes, and greater openness to using the product.

There were no significant differences on the levels of outcome variables at pretest based on gender, age, education, income, and geographical region, with the following exceptions. Women perceived e-cigarettes as more harmful than men did (5.2 vs. 4.5, *t*(450) = 2.60, *p <* .05). Among age groups, 18–29 year olds were significantly more open to trying e-cigarettes in the future than those 60 years old or older (1.66 vs. 1.08, *F*(3, 468) = 4.02, *p <* .01). In terms of geographical region, participants from the West perceived e-cigarettes as significantly more harmful than did participants from the Northeast (5.57 vs. 4.17, *F*(3, 476) = 4.23, *p* < .01).

### Effect of warning labels on changes in perceived harm

The GLM analyses on the effects of warning labels on changes in perceived harm for different tobacco products revealed significant time by group interactions for moist snuff (*F*(5, 473) = 2.54, *p* < .05) and e-cigarettes (*F*(5, 474) = 3.38, *p* < .01). There was no significant time by group interaction for snus (*F*(5, 471) = 0.49, *p* = .78).

**Figure 2:**
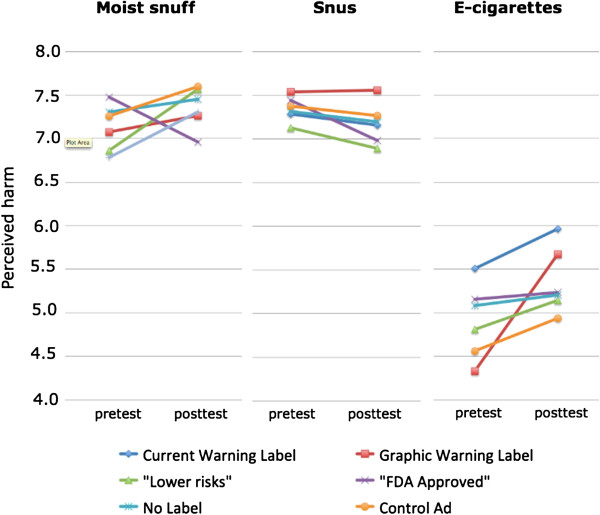
**Changes in perceived harm from pretest to posttest for cigarettes, snus, electronic cigarettes and moist snuff by warning label condition.** Note: Perceived harm was measured on a 9-point scale, with answers ranging from 1 – Not at all harmful to 9 – Extremely harmful.

Table 3 presents the differences in perceived harm from pretest to posttest. Seeing the advertisements with the current warning label increased perceived harm of e-cigarettes (*d* = 24). Exposure to graphic warning labels increased perceived harm of moist snuff (d = 0.27) and e-cigarettes (*d* = 0.54). Seeing the advertisement with “lower risk” label significantly lowered perceived harm of moist snuff (*d* = -0.24) and the “FDA Approved” label decreased perceived risk of snus (*d* = -0.25). The changes in perceived harm are represented graphically in Figure 2.

**Table 3:** **Effect of different warning labels and endorsements on perceived harm of tobacco products**

	Moist snuff			Snus			E-cigarettes		
	Pretest	Posttest	d	Pretest	Posttest	d	Pretest	Posttest	d
Current WL	7.08	7.26	0.10	7.29	7.16	-0.09	5.51	5.96	0.24*
Graphic WL	6.86	7.57	0.27*	7.54	7.56	0.01	4.33	5.67	0.54*
“Lower risks”	7.47	6.96	-0.24*	7.13	6.89	-0.12	4.81	5.15	0.15
“FDA Approved”	7.30	7.45	0.07	7.44	6.98	-0.25*	5.15	5.24	0.04
No Label	7.26	7.59	0.13	7.32	7.20	-0.05	5.08	5.20	0.05
Control Advertisement	6.78	7.31	0.20	7.37	7.27	-0.04	4.56	4.94	0.19

*p < .05 (Significant differences from pretest to posttest, multivariate simple effects of time based on the linearly independent pairwise comparisons among the estimated marginal means).

### Effect of warning labels on changes in positive attitudes towards alternative tobacco products

The GLM analyses for the effects of various labels on changes in positive attitudes towards alternative tobacco products showed no significant time by condition interactions for all three products: moist snuff (*F*(5, 465) = .402, *p* = .85), snus (*F*(5, 464) = .285, *p* = .92), and e-cigarettes (*F*(5, 467) = .736, *p* = .58).

For specific conditions (Table 4), advertisements with current warning label (*d* = -0.28) or graphic warning label (*d* = -0.26) decreased positive attitudes towards e-cigarettes significantly (this is a desired effect).

**Table 4:** **Effect of different warning labels and endorsements on positive attitudes towards alternative tobacco products**

	Moist snuff			Snus			E-cigarettes		
	Pretest	Posttest	d	Pretest	Posttest	d	Pretest	Posttest	d
Current WL	1.90	1.88	-0.02	1.93	2.02	0.07	3.06	2.67	-0.28*
Graphic WL	1.77	1.74	-0.03	1.84	1.85	0.00	3.48	3.02	-0.26*
“Lower risks”	1.76	1.72	-0.04	2.05	2.03	-0.03	3.68	3.56	-0.09
“FDA Approved”	1.75	1.60	-0.18	1.87	2.04	0.12	3.44	3.14	-0.20
No Label	1.45	1.51	0.09	1.72	1.76	0.03	3.39	3.11	-0.15
Control Advertisement	1.67	1.69	0.01	1.88	1.83	-0.03	3.78	3.69	-0.07

*p < .05 (Significant differences from pretest to posttest, multivariate simple effects of time based on the linearly independent pairwise comparisons among the estimated marginal means).

### Effect of warning labels on changes in openness to trying alternative tobacco products

For openness to trying alternative tobacco products, there were no significant time by condition interactions for all three product: moist snuff (*F*(5, 462) =1.84, *p* = .10), snus (*F*(5, 459) =1.40, *p* = .22), and e-cigarettes (*F*(5, 462) = 1.57, *p* = .17).

Openness to trying moist snuff went up in every condition (including control) with the exception of “lower risk” and no label condition where increases were not statistically significant (Table 5). For snus, openness increased in all but “lower risk” condition. For e-cigarettes, openness significantly increased in every condition except the graphic warning label and “FDA-approved” conditions.

**Table 5:** **Effect of different warning labels and endorsements on openness to trying alternative tobacco products**

	Moist snuff			Snus			E-cigarettes		
	Pretest	Posttest	d	Pretest	Posttest	d	Pretest	Posttest	d
Current WL	1.12	1.88	0.38*	1.08	1.75	0.39*	1.12	1.72	0.37*
Graphic WL	1.00	1.44	0.33*	1.03	1.34	0.29*	1.54	1.70	0.09
“Lower risks”	1.12	1.31	0.19	1.14	1.33	0.18	1.40	2.18	0.38*
“FDA Approved”	1.07	1.33	0.29*	1.15	1.47	0.26*	1.40	1.62	0.14
No Label	1.10	1.35	0.20	1.05	1.55	0.36*	1.15	1.71	0.42*
Control Advertisement	1.07	1.48	0.30*	1.05	1.33	0.24*	1.42	1.85	0.24*

*p < .05 (Significant differences from pretest to posttest, multivariate simple effects of time based on the linearly independent pairwise comparisons among the estimated marginal means).

### Behavioral sample selection

Overall, 14.9% (71) of participants were interested in receiving a free sample of alternative tobacco products, specifically, moist snuff (5.5%), snus (7.5%), and e-cigarettes (13.9%). There were no significant differences across conditions in the proportion of people interested in a free sample (*χ*^2^(5) = 1.95, *p =* .85). People who were interested in receiving a free sample of alternative tobacco products had significantly lower perceptions of harm of corresponding tobacco products (measured at posttest) than those not interested in a free sample. Specifically, those interested in a free sample of moist snuff (*M*int) had lower perceived harm of moist snuff (*M*int = 6.4) compared to those not interested (*M*not = 7.4; *t*(472) = 2.37, *p* < .05), for snus, *M*int = 6.1 vs. *M*not = 7.2 (*t*(463) = 2.74, *p* < .01), and for e-cigarettes *M*int = 3.8 vs. *M*not = 5.6 (*t*(97.3) = 5.79, *p* < .001). Unequal variance t-tests were used for e-cigarettes [32].

### Recall of warning labels

Correct recall of warning label differed by condition (χ^2^(5) = 33.48, *p <* .001), the highest recall being in “lower risks” (83.5%) and graphic warning label (80.2%) conditions, followed by control (correct recall of no warning label, 78.7%), then by current warning label (75.3%), “FDA Approved” (64.3%), and no warning label (47.1%). Only 2.5% of participants indicated seeing labels that were not used in the study.

## Discussion

To our knowledge, this research is the first to examine effects of an “FDA Approved” label and “lower risk” label for alternative tobacco products. Seeing advertisements with “FDA Approved” label significantly decreased perceived harm of snus among non-users of tobacco. Seeing advertisements with a “lower risk” warning label proposed by the R.J. Reynolds tobacco company (“Warning: No tobacco product is safe, but this product presents substantially lower risks to health than cigarettes”) significantly decreased perceived harm of moist snuff, the traditional smokeless tobacco product. This suggests that the endorsement label suggested by R.J. Reynolds may function similarly to the “FDA Approved” label, which is prohibited, decreasing perceived harm of tobacco products. Research using previously secret internal tobacco industry documents revealed that some tobacco companies have supported governmental regulation of tobacco as a strategy to improve their public image [33]. In doing so, tobacco companies framed tobacco use as an individual choice and portrayed themselves as responsible manufacturers of risky products [34]. The fact that the new warning label for smokeless tobacco proposed by R. J. Reynolds lowered perceptions of harm of moist snuff among non-users of tobacco indicates that this warning label applied to traditional smokeless tobacco (which has a relatively high level of perceived harm to begin with) can significantly lower perceptions of harm. Some argue that depicting smokeless and alternative tobacco products as less harmful than smoking will encourage smokers who cannot quit to switch to less dangerous tobacco products and avoid the worst health consequences of cigarette smoking [35, 36]. However, endorsing alternative tobacco products as less harmful might encourage initiation in youth or non-users, relapse in former smokers, or deter smokers from quitting, thus increasing harm to public health [37]. This study demonstrates that the “lower risk” warning label also leads to perceptions of lower risk among non-tobacco users. Even though participants in this study were non-smokers and non-users of smokeless tobacco, over 4% had tried one of the alternative tobacco products, most frequently e-cigarettes. Our data show that at least some non-smokers are trying and using e-cigarettes, and that interest in trying the products was higher among those non-users of tobacco who had lower perception of harm of these products. This study provides additional evidence suggesting the FDA should deny the R.J. Reynolds’s petition to replace the current warning label with a warning label communicating lower risk of smokeless tobacco.

This is also the first study to investigate the effects of warning labels on perceptions of electronic cigarettes. Placing current warning label used on smokeless tobacco (“Warning: This product is not a safe alternative to cigarettes”) or a graphic warning label on e-cigarette advertisements significantly increased perceived harm of e-cigarettes among non-users of tobacco products. It should be noted that perceived harm of e-cigarettes increased in all conditions (although not significantly) with the exception of when participants saw an e-cigarette advertisement without a warning label. In early 2014, the FDA proposed a “deeming rule” [38], extending its regulatory authority to other tobacco products, including electronic cigarettes. One of the provisions of this rule is to add the warning label to electronic cigarettes, “WARNING: This product contains nicotine derived from tobacco. Nicotine is an addictive chemical”. (If the product contains no nicotine, the proposed warning label would state, ‘This product is derived from tobacco”). Based on our findings, it is likely that any warning label on electronic cigarettes would increase perceptions of risk among non-users of tobacco, and, as such, would be helpful in reducing population-level harm by reducing interest in the use of the products among non-users of tobacco.

Given the current lack of evidence about the long-term health effects of e-cigarettes, warning labels could also communicate the uncertainty about health effects of e-cigarettes; future studies should examine the effects of these types of warning labels on perceived harm.

It is worth noting that although participants were non-users of tobacco (who had not smoked more than 100 cigarettes or used smokeless tobacco more than 20 times in their lifetime), almost 9% reported trying alternative tobacco products and over 4% were current (past 30 days) users (those current users were excluded from the study). E-cigarettes were used the most frequently with 5.3% reporting ever use and 3.4% reporting past month use, followed by snus (ever use 2.8%, past 30 days 1.0%) and moist snuff (ever use 2.6%, past 30 days 1.4%). These e-cigarette trial numbers are higher than those reported previously. In 2010, 0.8% of never-smokers ever tried and 0.3% reported past month use of e-cigarettes [39]; another report indicated 1.2% ever use among never smokers [40]. It appears that as electronic cigarettes are becoming more visible in the society, more non-smokers may be trying and using them.

We also examined effects of warning labels on attitudes and behavioral intentions and found little effect of specific warning labels on those variables. The only significant change across all conditions and all three products was that participants who saw either the current warning label or the graphic warning label significantly decreased positive attitudes towards e-cigarettes. These significant changes occurred in the same conditions that exhibited significant increase in perceived harm of e-cigarettes, suggesting that increase in perceived harm could possibly be responsible for decrease in positive attitudes towards e-cigarettes.

Openness to trying alternative tobacco products increased from pretest to posttest across every condition (significantly in over half the conditions) for all three products. Because the openness increased in every condition, even in control conditions where participants did not see advertisements for tobacco products, it is likely that this increase is an effect of a repeated testing. This finding is in line with a previous study (with a nationally representative sample of smokers) where openness to trying snus increased across all conditions [31]. It should also be noted that even with a slight increase in openness, participants predominantly remained “not at all open” at posttest, because the initial openness was so low, with most participants selecting the lowest possible option on the openness scale.

Although we did not measure actual behavior (e.g., tobacco use) in this study, we asked participants to select a free sample of the product and 14.9% of participants were interested in a free sample. There was no difference across the warning labels conditions, but overall, non-users of tobacco who wished to receive a free sample of alternative tobacco product held significantly lower perceptions of harm of these products than those who opted out of receiving free samples. Thus, perceived harm is related to potential behavior (trying the product), the finding that is in line with past research [10].

Recall for warning labels was different across the conditions, with highest recall for graphic warning labels. It should be noted that recall was only measured for the verbal portion – participants had to select among several textual warnings rather than indicate that they saw a graphic warning label. If we asked whether participant saw a graphic warning label, the recall might have been even higher. Low recall of “FDA Approved” label could be explained by the fact that people do not perceive it as ‘warning.’

Limitations of this study include that it was a non-probability-based sample; however, the sample was drawn from a diverse sample of non-users of tobacco in the United States with heterogeneous demographics. Our sample was more educated (with 58% of participants being college educated) as compared to the educational levels reported by the US census (29% of adult population in general, including tobacco users and non-users, being college educated) [41] and the 2012 NHIS (31% of non-smokers being college-educated) [42]. Although we did have a sizable proportion of lower educated participants and education was not related to our main outcome variables, the characteristics of the sample might suggest the study results may limit the generalizability of the study to more educated populations.

Self-reported measures of perceived harm, attitudes, and openness to trying tobacco products might have been subject to social desirability bias, resulting in the high ratings of perceived harm and low attitudes and openness levels that we observed in this study, underestimating the effects of “lower risk” and “FDA Approved” labels. While the increase in perceived harm of alternative tobacco products was unambiguous for the current and graphic warning labels, the “lower risk” and “FDA Approved” label conditions showed weaker effects, so larger samples may be needed to obtain stable and more conclusive estimates of these smaller effects. Future studies should take these issues into account.

## Conclusions

In conclusion, this study provides the first evidence against allowing “reduced harm” or “lower risk” labels on alternative tobacco products. While further data should be collected to validate our results, our findings provide initial evidence that endorsements such as the one proposed by RJ Reynolds may have similar effects to the prohibited “FDA Approved” label. Warning labels may be an effective way to decrease interest in e-cigarettes among non-users of tobacco. Regulatory agencies should consider implementing graphic warning labels for smokeless tobacco and investigate use of warning labels for e-cigarettes.
